# Anti-Nuclear Antibodies in Daily Clinical Practice: Prevalence in Primary, Secondary, and Tertiary Care

**DOI:** 10.1155/2014/401739

**Published:** 2014-03-13

**Authors:** Thomas Y. Avery, Mart van de Cruys, Jos Austen, Frans Stals, Jan G. M. C. Damoiseaux

**Affiliations:** ^1^Central Diagnostic Laboratory, Maastricht University Medical Centre, P. Debyelaan 25, 6229 HX Maastricht, The Netherlands; ^2^Department of Microbiology, Atrium Medical Centre, Henri Dunantstraat 5, 6419 PC Heerlen, The Netherlands

## Abstract

For the diagnosis of systemic autoimmune rheumatic diseases (SARD), patients are screened for anti-nuclear antibodies (ANA). ANA, as assessed by indirect immunofluorescence (IIF), have a poor specificity. This hampers interpretation of positive results in clinical settings with low pretest probability of SARD. We hypothesized that the utility of positive ANA IIF results increases from primary to tertiary care. We retrospectively determined ANA, anti-ENA, and anti-dsDNA antibody prevalence in patient cohorts from primary (*n* = 1453), secondary (*n* = 1621), and tertiary (*n* = 1168) care settings. Results reveal that from primary care to tertiary care, ANA prevalence increases (6.2, 10.8, and 16.0%, resp.). Moreover, in primary care low titres (70% versus 51% and 52% in secondary and tertiary care, resp.) are more frequent and anti-ENA/dsDNA reactivities are less prevalent (21% versus 39% in secondary care). Typically, in tertiary care the prevalence of anti-ENA/dsDNA reactivities (21%) is lower than expected. From this descriptive study we conclude that positive ANA IIF results are more prone to false interpretation in clinical settings with low pretest probabilities for SARD, as in primary care. Whether alternative approaches, that is, immunoadsorption of anti-DFS70 antibodies or implementation of anti-ENA screen assays, perform better, needs to be determined.

## 1. Introduction

The hallmark of autoimmune diseases is the pathologic activity of the immune system of an organism directed against its own cells and tissues. The disease is a direct consequence of tissue and/or organ damage as mediated by autoreactive components of the immune system, that is, autoreactive T-lymphocytes and/or autoantibodies. For diagnostic purposes, autoantibodies are the most important analytes. Within the systemic autoimmune rheumatic diseases (SARD), anti-nuclear antibodies (ANA), directed against various cellular components, and associated autoantibodies, such as antibodies reactive with dsDNA and extractable nuclear antigens (ENA), are fundamental for diagnosis [1–3].

Traditionally, ANA are detected by indirect immunofluorescence (IIF) performed on human epithelial cells (HEp-2). This technique requires a multistage process consistent with visual determination of the staining pattern, serial titrations of positive sera, followed by a second test in which autoantigen specificity is determined [2, 4]. Recently, the American College of Rheumatology (ACR) stated that ANA detection by IIF is still considered the gold standard [5]. This was primarily based on the high sensitivity of the IIF assay and the inclusion of ANA detection by IIF assay in diagnostic criteria of systemic lupus erythematosus (SLE) and autoimmune hepatitis (AIH) [6–8]. In addition, ANA can also be considered as a screening test for samples that require further testing for autoantigen specificity, that is, dsDNA and ENA [2, 9].

The specificity of ANA detection by IIF, however, is relatively poor, especially when low titres are used for screening. Indeed, at a 1 : 40 serum dilution, 25–30% of healthy individuals may test positive for ANA and this increases even further upon ageing [1, 10]. Overall, it is recommended that the serum dilution that gives a specificity of 95% in healthy individuals should be used as cut-off [3]. Moreover, the clinical significance rises with increasing titres [11, 12], as well as with the identification of the responsible autoantigen [1, 9]. Obviously, a positive ANA test must always be interpreted cautiously and only within the clinical context of the patient. In a clinical setting where the pretest probability of SARD is generally low, as in primary care, the added value of a positive ANA test is lower as compared to secondary and tertiary care situations where pretest probabilities of SARD are often higher [13].

In the current study, we determined the prevalence of ANA in primary (general practices), secondary (regional hospital), and tertiary care (university hospital). Besides data on ANA prevalence, also ANA titres and anti-ENA and anti-dsDNA antibodies were included in our analyses. We hypothesize that ANA prevalence, ANA titre, and anti-ENA/dsDNA reactivity increase from primary to tertiary care as these situations are expected to be also associated with an increasing pretest probability of SARD.

## 2. Materials and Methods

### 2.1. Patients/Participants

In the present study, three different patient populations from the southern part of The Netherlands were evaluated and compared with each other. These three populations consisted of patients who were tested for ANA between November 2011 and August 2012 in suspicion of an autoimmune disease. All ANA requests were considered to involve the diagnostic workup since none of the patients had requests for ANA (and/or anti-ENA/dsDNA) at least 4 years prior to the study period.

In the first patient population (*n* = 1453) ANA were requested by general practitioners (primary care). The second population (*n* = 1621) had an ANA request in a regional hospital (secondary care), while the third population (*n* = 1168) had an ANA request in a university hospital (tertiary care). Testing for ANA in the first and second cohorts was performed in the Atrium MC (Heerlen, The Netherlands), while the ANA tests in the third cohort were performed in the Maastricht University Medical Centre (MUMC, Maastricht, The Netherlands). Furthermore, in both regional and university hospitals, the origin, that is, hospital department, of ANA requests was documented.

### 2.2. Detection of ANA by IIF

ANA detection by IIF was performed on HEp-2000 cells according to the instructions provided by the manufacturer (Immuno Concepts, Sacramento, CA). Hep-2000 cells are transfected with the gene for SSA-60, which makes these cells more sensitive for SSA-antibody detection [14, 15]. Serum samples were screened in a 1 : 80 dilution. FITC-conjugated goat anti-human IgG antibody was used for detection of ANA. Five staining patterns were considered ANA positive: homogenous, atypical speckled, speckled, centromere, and nucleolar. In case of mixed-patterns, the pattern with the highest titre was included in the present study. Slides were evaluated with a fluorescent microscope (Axioskop, Carl Zeiss Microscopy GmbH, Jena, Germany) with LED light source. All slides were evaluated by two independent observers; in case of a difference in opinion, a third observer was decisive.

### 2.3. Detection of Anti-ENA Antibodies by LIA

The presence of anti-ENA antibodies was screened by a commercially available line immunoassay (ANA 3 Profile EUROLINE, Euroimmun, Lübeck, Germany). The assay was performed according to the manufacturer's instructions. The sera were diluted 1 : 100 in sample buffer. After the first incubation with diluted serum, a second incubation was performed with goat anti-human IgG linked to alkaline-phosphatase. Finally, a third incubation took place with bromochloroindolyl phosphate and nitro blue tetrazolium chloride (BCIP/NBT) as substrate to detect anti-ENA antibodies. Although the ANA 3 Profile EUROLINE kit enables detection of 15 different antigens, only eight were evaluated in the current study: Ro52, SS-A/Ro60, SS-B/La, nRNP/Sm, Sm, Scl-70 (topoisomerase 1), Jo-1, and CENP-B. Reading of the results was automated and the colour intensities of the reactions were evaluated by the EUROLineScan program (Euroimmun) to enable semiquantitative determination, that is, equivocal, 1+, 2+, and 3+. Results were considered positive for Sm, Scl-70, and Jo-1 if the intensity was at least equivocal, while for SS-A/Ro60, SS-B/La, nRNP/Sm, and CENP-B an intensity of at least 1+ was required. Finally, the cutoff for Ro52 was 2+.

### 2.4. Detection of Anti-ENA Antibodies by FEIA

Positive LIA results, as defined above, were confirmed with a commercially available FEIA (EliA, ImmunoDiagnostics, Thermo Fisher Scientific, Freiburg, Germany). This method uses highly purified (SmD) or recombinant (SS-A/Ro60, Ro52, SS-B/La, CENP-B, Scl-70, Jo-1, RNP70, and U1RNP) human antigens that are coated on irradiated polystyrene cups. The assay was performed according to the manufacturer's instructions. The sera were diluted 1 : 50 with dilution buffer. After binding of anti-ENA antibodies, the cups were washed and subsequently incubated with mouse anti-human IgG (heavy chain specific) conjugated to **β**-galactosidase. In case of antibody association, binding was detected fluorometrically using 4-methylumbellifery-**β**-D-galactoside (0.01%) as substrate. All assay procedures were fully automated in an ImmunoCAP250 (Thermo Fisher Scientific). The reference range was supplied by the manufacturer. For all antigens, values above 10 U/mL were considered positive.

### 2.5. Detection of Anti-dsDNA Antibodies by FEIA and CLIFT

In primary and secondary care, anti-dsDNA antibodies were detected with a commercially available FEIA (EliA, ImmunoDiagnostics, Thermo Fisher Scientific). This method uses a circular plasmid dsDNA, purified from* Escherichia coli*, as antigen. The assay was performed according to the manufacturer's instructions and as described for the anti-ENA antibodies. The sera were diluted 1 : 10 in dilution buffer and values above 15 U/mL were considered positive.

In tertiary care, anti-dsDNA antibodies were detected by the* Crithidia luciliae* immunofluorescence test (CLIFT; Immuno Concepts). Serum samples were screened in a 1 : 10 dilution. FITC-conjugated goat anti-human IgG antibody was used for detection of anti-dsDNA antibodies. Slides were evaluated with a fluorescent microscope (Axioskop, Carl Zeiss Microscopy GmbH) with LED light source. All slides were evaluated by two independent observers; in case of a difference in opinion, a third observer was decisive.

### 2.6. ANA/ENA Algorithm

All samples were tested first by ANA IIF. If the result was negative, no further testing was performed, unless specifically requested. However, the results of these additional tests were not included in the present study. If the ANA IIF was positive, irrespective of the staining pattern, titration was performed (1 : 320 and 1 : 1280). If a homogenous ANA pattern was detected, testing for anti-dsDNA antibodies was performed by FEIA (primary and secondary care) or CLIFT (tertiary care) [16]. Additionally, in case of a homogenous, (atypical) speckled, or centromere pattern, typing for anti-ENA antibodies was performed by LIA. Positive LIA results, as defined above, were tested by FEIA for confirmation. Confirmation was achieved if FEIA results were unequivocal positive. Because of the high correlation between the atypical speckled ANA pattern and SS-A/Ro60 antibodies [2], these antibodies were considered positive if the atypical speckled ANA pattern was observed in combination with either a positive LIA or a positive FEIA. Similarly, anti-CENP-B antibodies were considered positive when a centromere ANA pattern was observed in combination with a positive anti-CENP-B result in at least one of both anti-ENA antibody test systems. Since the LIA does not enable specific distinction of anti-RNP antibodies, positivity for the nRNP/Sm complex was followed by testing for antibodies against RNP70 and U-RNP by FEIA. Positivity of any of these two entities was interpreted as RNP positive. The same algorithm was applied to all requests within the 3 cohorts.

### 2.7. Statistical Analyses

All data analyses were performed using SPSS version 19.0 (SPSS Inc., Chicago, IL) or Graphpad Prism version 6 (Graphpad Software Inc., La Jolla, CA).

The Kolmogorov-Smirnov analysis was performed to determine whether the age distributions of the three study populations were normally distributed. Furthermore, the Chi square test, with Yates' correction if appropriate, was performed when comparing proportions of groups. In the case of small samples, Fisher's exact test was performed instead. A *P* value of <0.05 was considered statistically significant.

## 3. Results

### 3.1. Study Population and Origin of ANA Requests

Of the three patient populations included, 90 (6.2%), 175 (10.8%), and 187 (16.0%) patients from primary (*n* = 1453), secondary (*n* = 1621), and tertiary care (*n* = 1168), respectively, were tested positive for ANA and were therefore eligible for the current study. Gender (F/M) and age (median and range) distribution were as follows: 78/12 and 57.2 (15–95) for primary care, 129/46 and 57.0 (17–93) for secondary care, and 130/57 and 57.3 (3–84) for tertiary care.

The gender distribution differed significantly (*P* = 0.009) due to a strong female preponderance in primary care. The age distribution differed significantly (*P* = 0.005) due to the fact that in tertiary care age distribution was skewed negatively.

**Figure 1:**
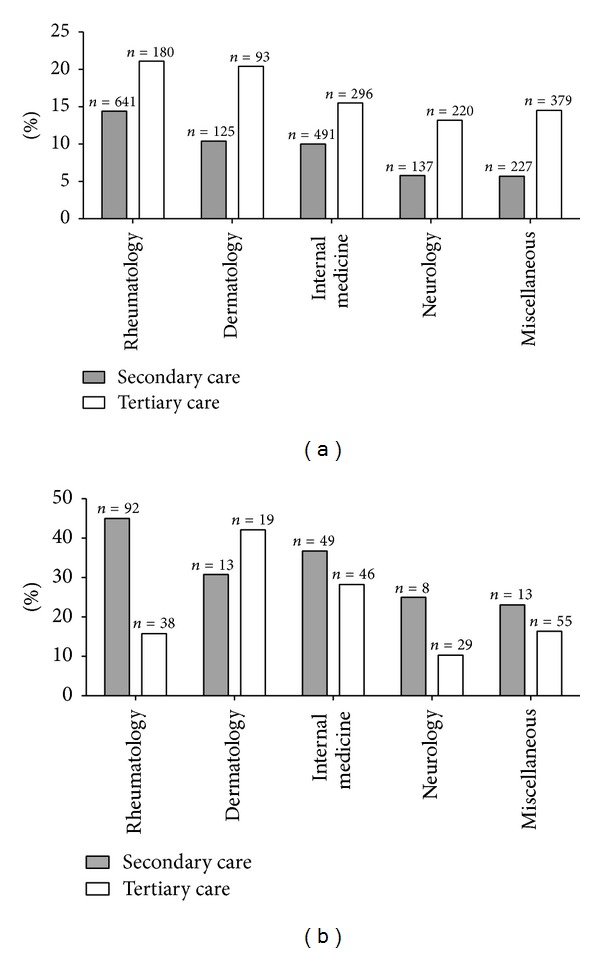
Relative presence of ANA and anti-ENA/dsDNA antibodies stratified for requesting clinical department. (a) The prevalence (%) of ANA positive sera in the rheumatology, dermatology, internal medicine, neurology, and miscellaneous departments of secondary (grey bars) and tertiary care (white bars) is displayed relative to the total amount of ANA requests per department (*n* above bars). (b) The prevalence (%) of anti-ENA/dsDNA reactivity in ANA positive sera in secondary (grey bars) and tertiary care (white bars) is displayed relative to the total amount of ANA positive sera (*n* above bars).

Evaluation of the origin, that is, hospital departments, of the ANA requests in secondary and tertiary care revealed four departments, that is, rheumatology, dermatology, internal medicine, and neurology, which requested the majority of the ANA screening tests (Figure 1(a)), that is, 86% and 68%, respectively. All other departments were collectively grouped as miscellaneous. This group was most diverse in tertiary care.

### 3.2. ANA Prevalence and Titre Increase from Primary to Tertiary Care

**Figure 2:**
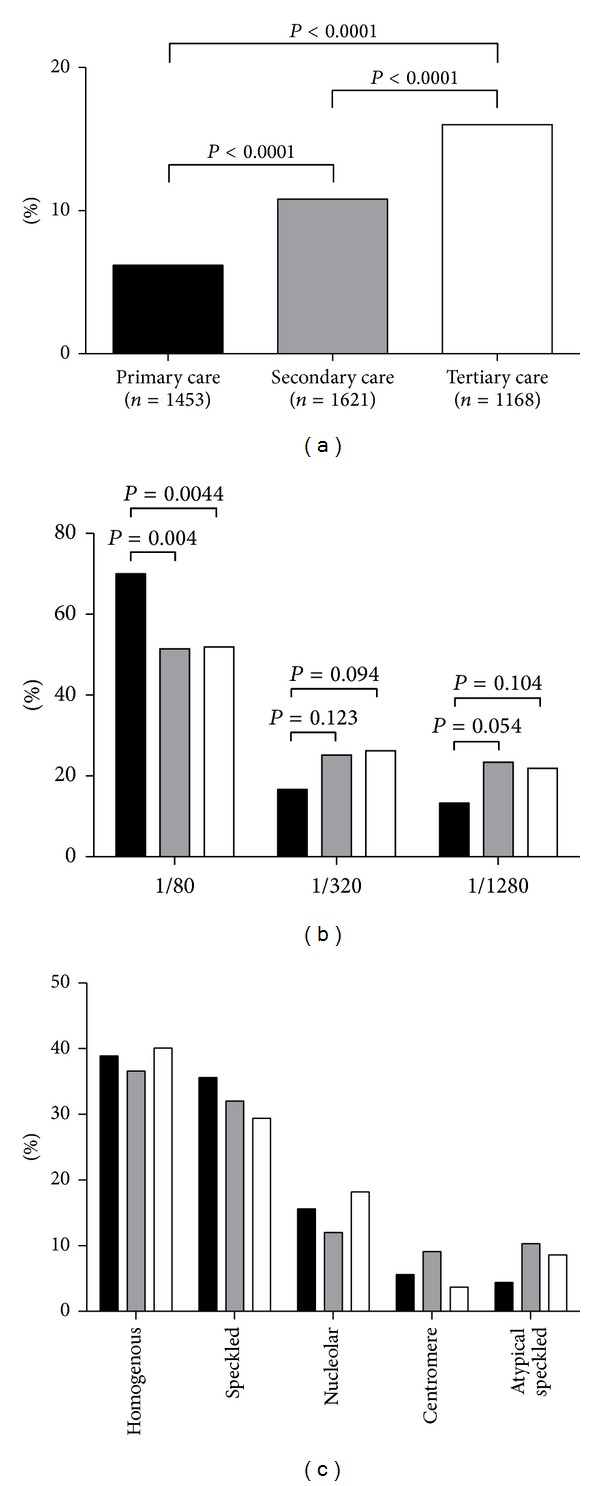
ANA reactivity stratified for primary, secondary, and tertiary care. The relative prevalence (%) of ANA positive sera is presented for primary, secondary, and tertiary care (a). The relative prevalence (%) of ANA titres (b) and patterns (c) is displayed in primary (black bars), secondary (grey bars), and tertiary care (white bars).

The prevalence of ANA in the 3 distinct clinical settings is depicted in Figure 2(a). The relative increase in the prevalence from primary to tertiary care is statistically significant. The higher relative ANA prevalence in tertiary care (16.0%) versus secondary care (10.8%) was apparent in all 4 clinical disciplines that requested the majority of ANA screening test (Figure 1(a)); this also holds for the other clinical disciplines (data only shown as pooled results). Typically, in secondary care 39.5% of overall ANA requests came from the rheumatology department, while in tertiary care this was 15.4%.

Within the positive ANA cohorts, patients from primary care had relatively low titres as compared to secondary and tertiary care (Figure 2(b)). Indeed, out of 90 positive ANA tests 63 sera (70%) revealed an ANA titre of 1 : 80. In secondary and tertiary care, a titre of 1 : 80 was obtained in 51.4% and 51.9% of the patients, respectively. Consequently, higher titres were observed more frequently in secondary and tertiary care than in primary care. The distribution in titres between secondary and tertiary care was not different.

At first glance, there is no apparent difference in the distribution of ANA patterns between the three health care levels (Figure 2(c)). Also, comparison of the distribution of ANA patterns with a titre of 1 : 1280, considered to have the highest positive likelihood ratio, showed no significant differences (data not shown).

### 3.3. Anti-ENA and Anti-dsDNA Antibodies Prevalence Is Highest in Secondary Care

**Figure 3:**
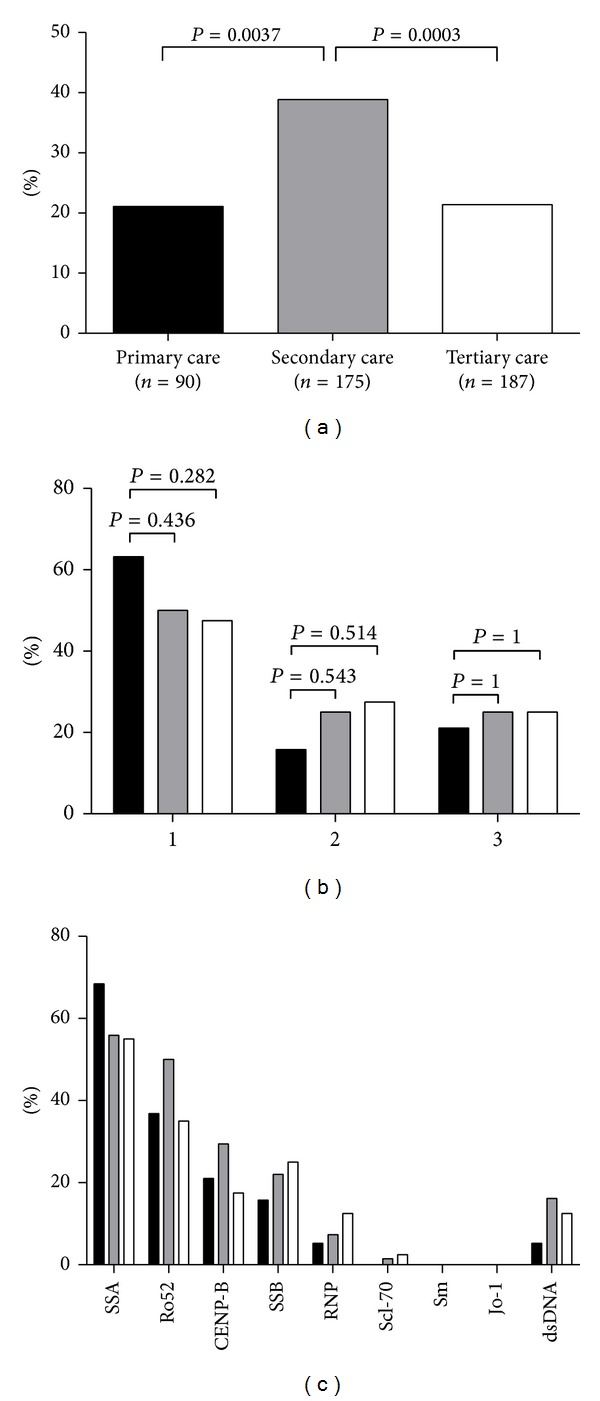
Anti-ENA/dsDNA reactivity stratified for primary, secondary, and tertiary care. The relative prevalence (%) of anti-ENA/dsDNA reactivity in ANA positive sera is presented for primary, secondary, and tertiary care (a). The relative prevalence (%) of 1 or more specificities (b) and type of specificities (c) is displayed in primary (black bars), secondary (grey bars), and tertiary care (white bars). Notably, in primary and secondary care anti-dsDNA antibodies were detected by FEIA, while in tertiary care CLIFT was the method of choice.

The prevalence of anti-ENA and anti-dsDNA antibodies, as defined by the algorithm described above, is presented in Figure 3(a). In the primary care, 19 (21.1%) patients with positive ANA (*n* = 90) were tested positive for anti-ENA and/or anti-dsDNA antibodies, that is, 1.3% of the total cohort. In the secondary and tertiary care, 68 of 175 (38.9%) and 40 of 187 (21.4%) ANA positive patients revealed anti-ENA and/or anti-dsDNA reactivity, respectively. This is 4.2% and 3.4% of the total secondary and tertiary care cohorts, respectively (*P* = 0.367). Significantly more positive anti-ENA and/or anti-dsDNA results were found in the total secondary and tertiary care cohorts than in the primary care cohort (*P* < 0.0001 and *P* = 0.0006, resp.). In secondary care, the prevalence of anti-ENA and/or anti-dsDNA reactivity within the positive ANA samples was the highest in the requests from rheumatology (Figure 1(b)). In tertiary care, however, departments of rheumatology and neurology had, on average, reduced prevalence of anti-ENA and/or anti-dsDNA reactivity as compared to the departments of dermatology and internal medicine (Figure 1(b)).

Since the relevance of anti-ENA and anti-dsDNA antibodies is considered to increase when combined reactivity is observed, we analysed the prevalence of anti-ENA and/or anti-dsDNA antibodies in relation to combined reactivity (Figure 3(b)). In the primary care setting, single positivity appeared to be more abundant than in secondary and tertiary care, but this difference was statistically not significant. No differences were observed between secondary and tertiary care settings either. With respect to the antigens recognized by the specific antibodies, no apparent differences were observed in the prevalence of antibodies reactive to RNP, SSA60, Ro52, SSB, CENP-B, and dsDNA (Figure 3(c)). Antibodies reactive to Sm and Jo-1 were not detected in any sample. Anti-Scl70 antibodies were only detected in samples of patients from secondary and tertiary care. However, the absolute number, one in each cohort, was low in both of these settings.

## 4. Discussion

In the present study on the analyses of ANA prevalence, ANA titre and anti-ENA specificity in the primary, secondary, and tertiary care, our results indicate that (i) ANA prevalence significantly increases from primary to tertiary care, (ii) low titres (1 : 80) are more frequently observed in the primary care, and (iii) anti-ENA and anti-dsDNA specificities are significantly more prevalent in the secondary care than in the primary care. Typically, the latter observation does not hold for the tertiary care.

Interpretation of the data obtained in the current study is highly dependent on the viewpoint on clinical utility of an ANA test result. It is tempting to start from the clinical utility of a positive ANA result. This result may help the clinician to identify a patient with SARD, but especially in situations with low pretest probabilities of such diseases, the risk of false interpretation of a positive ANA is high. This risk of false positive interpretation will decrease if the positive ANA is characterized by high titre and includes (multi)-reactivity for ENA and/or dsDNA, since these characteristics are associated with higher positive likelihood ratios [11, 12, 17]. Next, one has to realize that ANA testing is performed in the context of multiple diseases, varying from distinct SARD to autoimmune liver diseases. Interpretation of a positive ANA test may be different for each distinct disease: patients with, for instance, AIH or systemic sclerosis often have a positive ANA with no ENA reactivity, while a positive ANA test as such is part of the classification criteria for SLE, as well as AIH [6–8]. A negative ANA test result, on the other hand, may also be very useful to exclude a specific set of diseases and may drive attention to other diseases. Again, this differs for the distinct SARD: a negative ANA test has a high negative predictive value for SLE and systemic sclerosis but is less helpful to exclude Sjögren's syndrome or myositis [2, 3]. Obviously, definite interpretation of our dataset is hampered by the lack of clinical data from the patients of the three cohorts. The assumption of the primary care having a relatively low pretest probability was based on previous studies [13, 18, 19]. For future studies, we recommend the inclusion of clinical data in order to be able to thoroughly assess pretest probabilities and strengthen our assumptions. This study, however, also has noteworthy strengths, that is, only data obtained during the diagnostic workup of patients (not follow-up samples) were included and the same testing algorithm and reagents (except for detection of anti-dsDNA antibodies) were used for all three patient cohorts.

As expected, we observed a gradual increase in the prevalence of ANA from primary to tertiary care. In our present study ANA was detected by IIF in a screening dilution of 1 : 80. In several studies it has been reported that 10–15% of healthy controls are ANA positive in this serum dilution [1, 10, 20]. Obviously, a positive ANA test result is not only dependent on the dilution factor but also on the quality of other reagents, that is, fluorescent conjugate, cell substrate, and the fluorescent microscope [4]. For standardization purposes, it has, therefore, been recommended to screen for ANA with a specificity of 95% [3]. Taking this into account, the mere presence of ANA in our primary care cohort (prevalence 6.2%) lacks discriminating power to identify patients with SARD. On the other hand, due to the high negative predictive value of a negative ANA result, the general practitioner can reliably exclude certain diseases. Furthermore, since both high titre and anti-ENA and/or anti-dsDNA (multi-)reactivity may increase the likelihood of identifying a patient with SARD [11, 12, 17], it is apparent in our primary care cohort that 30% of ANA positive sera are of medium to high titre (*n* = 27), 21% contain anti-ENA and/or anti-dsDNA reactivity (*n* = 19), and 37% of the latter reveal multireactivity (*n* = 7). In our secondary and tertiary cohorts, the ANA prevalence is also relatively low (11 and 16%, resp.), but in both clinical settings about half of the ANA positive sera are of high titre (48-49%). As expected, the observed anti-ENA and/or anti-dsDNA reactivity of ANA positive sera in the secondary care (39%) is higher than in the primary care. Surprisingly, anti-ENA and/or anti-dsDNA reactivity in the tertiary care (21%) is lower than in the secondary care and similar to the primary care. This might be related to the spectrum of diseases investigated in the tertiary versus the secondary centres. Also the higher number of ANA requests by tertiary care departments not typically involved in the diagnosis of SARD might be the result of academic profiling of the respective departments. For instance, the cardiology department of the MUMC is specialized in inflammatory cardiomyopathies and a possible autoimmune aetiology of these diseases. Cardiology requested 108 ANA tests (9.2%), revealing 13% ANA positive results of which 20% was anti-ENA and/or anti-dsDNA antibody positive. A second important difference is the presence of a division of clinical immunology within the department of internal medicine of the tertiary care hospital, implying that many patients with SARD are evaluated by clinical immunologists instead of rheumatologists. The latter difference might at least explain the lower relative prevalence of anti-ENA and anti-dsDNA antibodies in the rheumatology department of the tertiary care hospital.

Our study shows that in secondary care the majority of positive ANA results (92.6%), as well as the positive anti-ENA and anti-dsDNA results (95.6%), are linked to the departments of rheumatology, dermatology, internal medicine, and neurology. Obviously, patients with the highest pretest probabilities for SARD, as associated with the initial clinical presentation, are most likely referred to these clinical departments. In clinical settings with lower pretest probabilities, ANA positive sera are more likely to be of no clinical significance [13, 18, 19]. It might therefore be an option, in particular in primary care settings, to move away from the traditional ANA screening test. In this context, the recent discovery of the dense fine speckled (DFS70) antigen is promising and could offer a possible solution for the identification and exclusion of positive sera with no clinical relevance [21, 22]. The typical ANA dense fine speckled pattern (DFS), known to be associated with the DFS70 antigen, has been found to be commonly prevalent in healthy individuals with ANA positive sera (33.1%), whereas in SARD patients 0.0% of the sera revealed a DFS pattern [22]. Another study revealed that 2-3% of SARD patients had antibodies directed to the DFS70 antigen [23]. Obviously, in SARD patients other anti-ENA antibodies might be present that hamper correct recognition of the DFS IIF pattern. Indeed, the identification and correct interpretation of the DFS pattern might prove to be challenging for diagnostic laboratories and would require additional training [24]. The laboratories involved in the current study did not distinguish the DFS pattern. Moreover, the majority of patterns recognized do not seem to be compatible with a DFS pattern, but this might be the consequence of only interpreting the strongest pattern in the current study. Since the identification of the DFS pattern might be challenging for routine diagnostic laboratories and inaccurate interpretation can have significant consequences [25], an immunoadsorption protocol to diminish anti-DFS70 antibody reactivity to HEp-2000 cells could be implemented in the current IIF assay in order to significantly improve the performance characteristics of the ANA IIF test [21, 25]. This approach sustains recognition of SARD-related autoantibodies in sera with combined reactivities, that is, anti-DFS70 and other anti-ENA antibodies. Another alternative testing algorithm could, instead of ANA IIF, include solid phase assays including multiplex and screening assays for well-defined anti-ENA and anti-dsDNA reactive antibodies [11, 12, 26, 27]. Both approaches enable to distinguish or reduce the number of positive ANA results lacking clinical relevance. In addition, multiplex anti-ENA screening assays are considered to better recognize particular antigens, for example, SSA and Jo-1, as compared to ANA IIF testing [3, 11, 12, 28].

Altogether, the results indicate that in the primary care the usage of traditional ANA screening tests is more prone to false-interpretation of positive ANA results. Rather, an alternative testing algorithm for detection of patients with SARD might be more appropriate. This might either be achieved by immunoadsorption of anti-DFS70 antibodies or direct screening for anti-ENA antibodies. Obviously, a patient with severe clinical manifestations typical for SARD presenting in a primary care setting should be referred directly, that is, without any laboratory testing, to the rheumatologist or clinical immunologist. This recommendation may not only apply for general practitioners but may also hold for clinical departments that are less likely to encounter patients suspected of SARD.
